# Perceived Quality of Postoperative Handover by Saudi Nurses: A Single-Center Cross-Sectional Study

**DOI:** 10.7759/cureus.43845

**Published:** 2023-08-21

**Authors:** Somayah Mohsen Mohammed Al-Qarni, Hala Mohamed Mohamed Bayoumy, Dalyal Alosaimi

**Affiliations:** 1 Intensive Care Unit, Prince Mishary Bin Saud Hospital, Al Baha, SAU; 2 College of Nursing, Cairo University, Cairo, EGY; 3 Department of Nursing, Vision College of Dentistry and Nursing, Riyadh, SAU; 4 College of Nursing, King Saud University, Riyadh, SAU

**Keywords:** handover quality, perception, nursing, handover, postoperative

## Abstract

Background: Handover is considered a basic nursing practice in which a patient's care information is moved to another nurse. Handover of patients after surgery is critical due to a number of care transitions, the presence of a surgical procedure, and the influence of anesthesia. High-quality postoperative handover is essential to safe patient care. Few studies have been conducted to evaluate the quality of current postoperative handover practices and the factors contributing to the quality of such processes, especially in Saudi Arabia.

Aim: The present research aimed at evaluating nurses' perceptions of postoperative handover quality and assessing factors impacting this process. This cross-sectional study targeted registered nurses with at least one year of professional experience who were actively involved in the conduction of postoperative handovers across various surgical departments. A total sample of 143 nurses was selected via a convenient sampling technique. Study instruments included Handover Quality Rating Form, patient status, and nurses’ background characteristics.

Results: Overall, postoperative handover quality was perceived as high by handing over and receiving nurses. Generally, 55.2% of nurses agreed on the different items supporting the positive circumstance for handover, and 92.3% agreed on the good conduct of handover compared to only 7.69% disagreement (p˂0.001). Significant agreements were observed for teamworking (p˂0.001), as well as four indicators (out of five) measuring the overall handover quality (p<0.001). The type of involved departments impacted significantly the handover quality perception (p=0.004). The respondents' age had a significant effect on quality (p=0.036), as well as circumstances of postoperative handover (p=0.046). Moreover, significant statistical differences were found for the circumstance of handover (p=0.031), as well as teamwork (p=0.019) according to the nurses' roles. Finally, the patient's blood circulation and respiration had a significant effect (p=0.023, p=0.033, respectively), as did the patient’s level of consciousness (p=0.006) in the nurses’ perception of the overall postoperative handover quality.

Conclusion: Postoperative handover quality was highly perceived by nurses. This research explored a multitude of factors such as patient health status and nurses’ socio-demographic variables and their impact on nurses’ perception of handover quality. Several nurse and patient-related factors were found to impact the handover process. This current research provided findings that could direct future improvements in nursing handover practice to ensure high-quality patient care.

## Introduction

Nursing handover is a process of transferring patients among different hospital departments, which requires comprehensive communication of the needed information to ensure the continuity of patient care [1]. According to the National Institute of Health and Care Excellence (NICE) [2], nursing handover is a system by which the responsibility for continued and urgent patient care is shared across various departments of the healthcare organization. The World Health Organization (WHO) [3] identified nursing handover as the communication process by which information specific to the health of a patient is shared between a team of caregivers or between caregivers and patient or family members, to comply with standards of care continuity and safety practices. Such definitions not only demonstrate how common or essential is the practice of handover in the nursing profession but also demonstrate the importance of ensuring that a patient receives high-quality care [4].

A clinical handover that is comprehensive and safe is essential for optimum patient care and prevention of clinical errors [5]. Caution has to be taken to ensure that there lies consistency and patient-centeredness in communicating the interventions, diagnostic needs, and changing health status of the patients during their time of stay in the healthcare facility [6]. Such consistency is necessary to ensure that patient care is not compromised irrespective of changes in individuals and environments providing the needed care [7]. Differences in handover communication components can exist in the form of "incorrect or incomplete information, wrong diagnosis, incomplete medical history or intolerance, lack of information on the history of care, limited or incorrect information on the surgical procedures, and diagnostic tests or medication administration factors (dosage, time, type)" [8,9].

In fact, the practice of handover can further be prone to differences in quality standards pertaining to documentation of critical patient health status, treatment needs, and medical history variables [10]. Postoperative handover occurs between hospital surgical departments that involve nurses with different experiences, expectations, perceptions, and patients with different conditions. Additionally, postoperative handover, being a complex process with multitasks such as vital signs monitoring and patient transferring, raises the chances of error [5]. The resulting loss of quality and ineffectiveness of clinical handovers were found to be a reason for the most significant adverse events, healthcare errors, and poor health outcomes among patients according to the reviewed root cause analyses and sentinel events [11-13]. In Saudi Arabia, out of 433 reported sentinel incidents between 2012 and 2015, 35% of them were due to failure in communication [14]. Identifying the potential risks associated with handover practices is likely to improve timely correction, resulting in improved handover and patient care quality [9,15,16].

Healthcare organizations should therefore ensure that all handovers comply with a "minimum dataset" irrespective of departmental differences. The minimal amount of information required to provide patient care and address health status and risks should be communicated sufficiently during interdepartmental transfer. Horwitz et al. [17] have discovered five factors that govern the quality of a handover, including the acquaintance with the patient, the number of handovers to be conducted, a sense of duty and responsibility, the presence of a leader, and a documented handover sheet. Healthcare organizations thus have been advised to follow a standard structured format of handover communication while transferring patients from one department to another [2,18]. The structured and standardized handover should include all content and information necessary for the continuity of care to patients [1]. Several studies have therefore focused on the importance of using a standardized handover process after surgery and indicated the positive impact of such standardization [11,19].

NICE [2] emphasized that following a structured handover tool or format is one of the most effective ways to prevent errors associated with the subjective nature of interpreting written or verbal handover information. Care transition processes primarily occur during the transference of handovers, primarily between practitioners possessing diverse skills, training, competencies, knowledge, expectations, and perspectives of care. It is worth mentioning that of all sentinel incidents that occurred during the transition of patient care in the hospital’s environment, 80% were due to poor communication among healthcare professionals [20-22]. Communication and cooperation are therefore important in enabling an effective handover process procedure [17,19]. Weinger et al. [23] postulated that effective communication processes are of utmost importance for maintaining patient safety.

However, there is evidence of many variations in the way nurses share, transfer, and receive communication about postoperative patient handovers, resulting in various styles and tools used for the process [24]. Mohammad [25] in his analysis of 80 nursing interactions, conducted across seven diverse wards in two hospitals in Saudi Arabia, revealed that linguistic differences influenced nursing interactions during the handover process. A systematic review conducted by Smeulers et al. [9] identified several different handover styles. These comprised verbal handovers where nurses share patient information by reading out clinical notes of the patient, the combined practice of verbal communication as well as reading aloud and taking down clinical notes, or the practice of handover communication at the patient’s bedside to allow for patient participation.

Additionally, Reine et al. [5] reported some other key factors that were found to be associated with a poor quality of nursing handover. These included uncertainty of clinical situations, high frequency of situations with time constraints, and pressures and the complexity of health problems experienced by patients. Several other barriers to effective handover processes were also communicated in the narrative literature review by Suganandam [26]. Such barriers include the resistance shown in response to changes such as the practice of standard tools for handover communication, limited time by nurses, limited resources to evaluate the existing quality of handover processes, inadequate levels of knowledge held by staff, the bad ratio of staffing between patients and nurses, the lack of leadership, the prevalence of cultural differences between nurses, and an absence of technology used to share patient information between one department to another. Several factors therefore can cause transferring a weak or incomprehensive handover between departments. Hence, nurses need to be aware of the various factors affecting handover quality and take appropriate actions to improve the process [27].

To sum up, differences in the quality of a handover are likely to raise the risk of clinical errors [5], especially in a postoperative patient who is characterized by the vulnerability to develop complications due to the surgical procedure and the effect of anaesthesia as well as the multiple transitions between hospital departments [11]. Thus, it is important to understand the perceptions held by nurses concerning the quality of handover procedures and practices to identify the areas that can be further enhanced for future nursing practice. However, according to the best of the researchers’ knowledge, no studies were previously conducted to assess the quality of the postoperative patient handover practice in Saudi Arabia. Hence, in this respect, the present study was undertaken to evaluate the postoperative patient handover quality among both the transferring and receiving nurses. Likewise, there was a shortage of existing literature in exploring the impact of nurses’ socio-demographic characteristics on the present differences underlying perceptions of handover quality in hospitals. Gaining knowledge of the postoperative handover practices and their influencing factors is important for nursing administration and policymakers to continually plan and implement appropriate interventions. Thus, this research also provided findings that could direct future improvements in nursing handover practice to ensure high-quality patient care, as well as positive health outcomes across the population residing in Saudi Arabia.

## Materials and methods

Research aim

The present research aimed to evaluate nurses' perceptions of postoperative handover quality among receiving and transferring nurses in a tertiary hospital in Saudi Arabia. Moreover, factors impacting the handover quality were investigated, including involved departments, nurses’ demographic and background variables, and finally patient status.

Research design

A descriptive, cross-sectional research design was carried out. Since this research aimed to understand how consideration of factors such as nurses’ roles, the status of patients, and nurses’ background variables influence perceptions concerning the quality of the nursing handover, a descriptive form of research was most appropriate to obtain in-depth knowledge and understanding of the issue or phenomenon under study [28].

Research setting and population

This research was carried out at King Khalid University Hospital (KKUH), one of the largest tertiary healthcare organizations situated in Riyadh, Saudi Arabia, with a capacity of 850 beds.

The population frame based on which the sample of the present research was drawn was the registered nurses who are carrying handing over or receiving postoperative patients between surgical departments of the selected hospital. Therefore, the total population for the present study was 281 registered nurses. A convenience sampling strategy was implemented.

An overall sample size of 143 registered nurses was calculated using the sample calculator of Raosoft, with a 95% confidence level and a 5% margin of error. The researchers ensured that the sample met the following inclusion criteria: registered nurses with at least one year of professional experience and being employed in departments including the operating room, post-anesthesia care unit (PACU), surgical intensive care unit, day surgery unit (DSU), or surgical inpatient wards. As data collection was carried out during the COVID-19 pandemic, there was absolutely limited access specifically to those wards that contained the high-risk group of postoperative pediatric, neonate, or maternity patients and also their nursing staff. Exclusion criteria, therefore, involved nurses who were receiving or transferring postoperative pediatric, neonate, or maternity patients, as well as nurses with problems handling English questionnaires.

Data collection instruments

The self-reported questionnaire, comprising three sections, was utilized for data collection. The first section included questions related to the patient’s age and medical status with respect to the following variables: blood circulation and respiration (impaired, medium, good), level of patient’s consciousness (unconscious, semiconscious, awake), feelings or experiences of the patient concerning wellness or nausea (no or yes), pain perceptions in patients (strong pain, moderate, slight, no pain), surgical procedures used, and anesthetic techniques. Additionally, information was collected concerning the physical status classification of the patient as per the American Society of Anesthesiology.

The second section contained information regarding the nurse participants’ background characteristics, including the total years of professional experience in practicing nursing, the nurses' age, professional experience in the concerned department, educational level, and current nursing roles. This section of the questionnaire further involved collecting data pertaining to handover areas and nurses' roles (whether transferring nurses or receiving nurses). The last section of the questionnaire involved the Handover Quality Rating Form (HQRF), formulated by Manser et al. [29]. The HQRF included 21 items focused on evaluating the overall handover quality. These items were divided into four dimensions that included circumstances of handover (four items), the conduct of handover (eight items), teamwork (four items), and handover quality (five items). Individual items for each dimension can be found in the Results section (Tables 2-5). Each item was rated on a four-point rating scale, ranging from agreeing (4), partially agreeing (3), partially disagreeing (2), to disagreeing (1) [29]. For each dimension, a mean score was calculated using its related sub-items scores. This mean score was further utilized to calculate the percentage agreement/disagreement for that construct using the four-point rating reference. All questionnaires in the three sections were in English. No attempts were done for Arabic translation as the English language is the primary language in nursing education and in many hospitals in Saudi Arabia.

Pilot study

It is worth denoting that the HQRF was developed and validated by Manser et al. [29], from whom the researchers had obtained permission via mail. A pilot study was carried out, using 20 registered nurses, to verify the validity and reliability of this instrument and to identify necessary modifications. The pilot study sample was excluded from the main study.

The validity was evaluated using Pearson's correlation coefficient. The statistical analysis indicated a statistically significant correlation at the level of α = 0.05 between the scores of each item and the total score of the dimension to which it belongs. Circumstances of the handover item correlations ranged between 0.769 and 0.851, the item correlation for the conduct of the handover ranged between 0.520 and 0.735, the item correlation for teamwork ranged between 0.598 and 0.866, and finally the item correlation for handover quality ranged between 0.467 and 0.783.

 Moreover, to verify the reliability of the HQRF, Cronbach's alpha was calculated. The values ​​of stability on the dimensions of the tool showed scores ranging between 0.702 and 0.814, which indicated a high degree of stability.

Data collection process

The researchers ensured that the design and methods are ethically compliant and respectful of the recruited participants' dignity, expectations, safety, and possibility of risk. Ethical approval was obtained from the Institutional Review Board of King Saud University, a notable ethics community of the university (#E-20-5456), and ethical approval was provided by the KKUH from where participants of the study were recruited [30,31].

To ensure that this research is respectful of the autonomy of the participants, the researchers ensured that informed consent was obtained and that participants were informed that their recruitments were voluntary and free of coercion [32]. The researcher ensured that each participant was well-informed concerning the present research purpose and procedures. Furthermore, participants were assured that they have the freedom to withdraw from the study and that participation will not cause any risk or harm to their healthcare whatsoever [33].

All nurses that met the inclusion criteria were invited to participate in the study. Upon securing the necessary approval for participation, eligible participants were requested to complete the handover quality questionnaire. Data collection was carried out between February 2021 and March 2021 using electronic devices (iPad) due to the COVID-19 precautions. To maintain privacy and confidentiality, participants’ sociodemographic characteristics and patient status information were kept anonymous without any identification, and data were secured in a safe database with a password and limited access to only researchers and authorized stakeholders from IRB [34].

Data analysis

This study utilized the Statistical Package for Social Science (SPSS) version 23.0 (IBM, SPSS Inc., Chicago, IL, USA) for statistically analyzing the collected data. To assess for statistically significant differences in the perception of postoperative handover quality regarding patient and socio-demographic variables and between receiving and transferring nurses, chi-square, goodness-of-fit, independent sample T-test, and one-way ANOVA tests were conducted. Posthoc analyses were carried out using the Bonferroni test for analyses that had omnibus (i.e., initial ANOVA) significant differences. In this study, the significant level (p-value) was set at <0.05.

## Results

Demographic profile of the study subjects

A total of 143 nurses voluntarily participated in the present study. From Table 1, most respondents (63%, n= 90) had six to 15 years of nursing experience. The majority of the respondents (57.3%, n=82) had an age range of 21-40 years.

**Table 1:** Demographic Profile of the Nurse Participants (n=143)

Variable	Group	Frequency	Percent
Total professional experience	1-5	21	14.7%
	6-10	43	30.1%
	11-15	47	32.9%
	16-20	18	12.6%
	>20	14	9.8%
Age of the nurse	24-30	38	26.6%
	31-40	82	57.3%
	>40	23	16.1%
The experience in the present unit	1-5	66	46.2%
	6-10	57	39.9%
	>10	20	14.0%
Educational level	Diploma	35	24.5%
	Bachelor or higher	108	75.5%
Current nursing role (position)	Staff nurse	118	82.5%
	Charge nurse	25	17.5%

The majority of respondents (75.5%, n=108) had a bachelor's degree or higher degree, while 35 nurses (24.5%) had a diploma. More than half of the nurse participants (82.5%, n=118) were staff nurses, and 17.5% (n=25) were charge nurses.

Eighty-four percent of the conducted surgical procedures were elective, while only 16% were done as emergency surgery. Moreover, 64.34% of the patients were younger than 50 years old, while 35.66% were older than 50 years old. Furthermore, 47.79% of the procedures were general surgeries, and 24.26% were orthopedic surgeries.

Overall postoperative nursing handover practice quality

Table 2 shows the responses of nurses on each statement related to the circumstances of the handover. The majority of the respondents (101, 70.6%) agreed that the case handed over did not involve high uncertainty versus (42, 29.4%) who agreed (chi-square=24.34, p˂0.001). The rest of the items did not show any significant statistical differences. Generally, 55.2% of nurses agreed on the different items supporting the positive circumstance for handover, while 44.7% disagree on the circumstance of handover (p˂0.001), indicating that the circumstances of postoperative handover were satisfactory and almost perceived well.

**Table 2:** Nurses’ Evaluation of the Circumstances of the Postoperative Handover

Item	Statement	Agree/Partially agree (%)	Disagree/Partially disagree (%)	Chi-square (p-value)
Q1	The person handing over the patient was not under time pressure.	67 (46.9%)	76 (53.1%)	0.57 (0.452)
Q2	The person taking on the responsibility of the patient was not under time pressure.	71 (49.7%)	72 (50.3%)	0.01 (0.933)
Q3	The case that was handed over was not of high complexity.	79 (55.2%)	64 (44.8%)	1.57 (0.210)
Q4	The case that was handed over did not involve high uncertainty.	101 (70.6%)	42 (29.4%)	24.34(˂0.001)
Circumstances of the handover		79 (55.2%)	64 (44.7%)	19.57 (˂0.001)

Table 3 illustrates the response of participants to the conduct of the handover. More than three-quarters of nurses agreed with the eight items as shown. In short, the results reflected the satisfactory performance of the postoperative handover. There was 92.3% agreement on all statements compared to only 7.69% disagreement (chi-square=99.807, p˂0.001). The majority of nurses agreed that the handover followed a logical sequence (Q1:139, 97.2%), the person handing over continuously used the available documentation (Q2:141, 98.6%), attempts were made to minimize the interruptions during handover (Q4:124, 86.7%), all relevant information were communicated (Q5:141, 98.6%), priorities for further treatment were addressed (Q6:139, 97.2%), the patient assessment data were properly communicated (Q6:140, 97.9%), and that possible risks and complications were discussed (Q8:136, 95.1%).

**Table 3:** Nurses’ Evaluation of the Conduct of the Postoperative Handover

Item	Statement	Agree/Partially agree (%)	Disagree/Partially disagree (%)	Chi-square (p-value)
					Q1	The handover followed a logical structure.	139 (97.2%)	4 (2.8%)	127.45 (p˂0.001)
Q2	Available documentation used to structure the handover.	141 (98.6%)	2 (1.4%)	135.11 (p˂0.001)
Q3	Enough time was allowed for the handover.	110 (76.9%)	33 (23.1%)	41.46 (p˂0.001)
Q4	Attempts were made to minimize interruptions during the handover.	124 (86.7%)	19 (13.3%)	77.10 (p˂0.001)
Q5	All relevant information was selected and communicated.	141 (98.6%)	2 (1.4%)	135.11 (p˂0.001)
Q6	Priorities for further treatment were addressed.	139 (97.2%)	4 (2.8%)	127.45 (p˂0.001)
Q7	The person handing over the patient clearly communicated his/her assessment of the patient.	140 (97.9%)	3 (2.1%)	131.25 (p˂0.001)
Q8	Possible risks and complications were discussed.	136 (95.1%)	7 (4.9%)	116.37 (p˂0.001)
Conduct of the handover		132 (92.3%)	11 (7.69%)	99.807 (p˂0.001)

Table 4 displays the four assessment items related to teamwork during postoperative handover. Almost all participating nurses agreed on items Q1 and Q4. Only one respondent (0.7%) disagreed about easiness to establish satisfactory contact at the beginning of the handover. The majority of nurses (n=120, 83.9%) agreed that there was no tension between the teams during the handover, and 138 respondents (96.5%) agreed that questions and ambiguities were resolved. Significant statistical differences were observed between agreement and disagreement on items Q1, Q2, and Q3, as well as on teamwork as a whole (p˂0.001).

**Table 4:** Nurses’ Evaluation of the Teamwork of the Postoperative Handover

Item	Statement	Agree/Partially agree (%)	Disagree/Partially disagree (%)	Chi-square (p-value)
					Q1	It was easy to establish good contact at the beginning of the handover.	142 (99.3%)	1 (0.7%)	139.03 (p˂0.001)
Q2	No tension between the team during the handover.	120 (83.9%)	23 (16.1%)	65.8 (p˂0.001)
Q3	Questions and ambiguities were resolved.	138 (96.5%)	5 (3.5%)	123.7 (p˂0.001)
Q4	The team jointly ensured that the handover was complete.	143 (100%)	0 (0%)	Cannot be calculated
Teamwork		122 (85.31%)	21 (14.69%)	50.09 (p˂0.001)

Table 5 shows the five indicators for the overall handover quality. Significant statistical differences were observed between agreement and disagreement on four items (Q1, Q2, Q3, and Q5), as well as on the handover quality as a whole (p<0.001). Regarding documentation, 97.9% of respondents reported agreement that the documentation was complete. More than half of the experienced nurses (59.4%) agreed that too much information was given and asked for during the handover (i.e., they disagreed with the statements in Q2 and Q3). Interestingly, 115 respondents, representing 80.42% of the total sample, rated the postoperative handover as of high quality.

**Table 5:** Nurses’ Evaluation of the Handover Quality of the Postoperative Handover

Item	Statement	Agree/Partially agree (%)	Disagree/ Partially disagree (%)	Chi-square (p-value)
Q1	Documentation was complete.	140 (97.9%)	3 (2.1%)	131.25 (p˂0.001)
Q2	Not too much information was given.	58 (40.6%)	85 (59.4%)	5.1 (0.024)
Q3	Not too much information was asked for.	59 (40.6%)	86 (59.4%)	5.1 (0.024)
Q4	The patient’s experience was considered carefully during the handover (respect).	143 (100%)	0 (0 %)	Cannot be calculated
Q5	Overall, the quality of this handover was very high.	131(91.6%)	12 (8.4%)	99.03 (p˂0.001)
Handover quality		115 (80.42%)	28(19.58%)	282.899 (p˂0.001)

In summary, the postoperative handover was conducted well under ideal circumstances, with satisfactory teamwork and high-rated quality.

Nurses’ perception of the postoperative handover quality based on involved departments

For testing group differences, the mean score for each item was calculated using the nurse participants’ ratings. Moreover, the HQRF dimensions' mean score was calculated by summing up the subitem mean scores and dividing the result by the number of items under each dimension. These two calculated scores were utilized in carrying out the comparison of means using the t-test and ANOVA tests.

ANOVA test was used to determine the differences in nurses’ perception of the postoperative handover quality as per postoperative involved departments. Results in Table 6 revealed no significant statistical difference in the agreement on the circumstance of handover (p>0.05), the conduct of handover (p=0.576), or teamwork (p=0.106) based on the involved postoperative handover departments.

**Table 6:** Nurses’ Rating of the Overall Postoperative Handover Quality Based on the Involved Areas (From-To) SD=Std. Deviation; μ=Mean; OR=Operating Room; PACU=Pediatric Intensive Care Unit; SICU=Stepdown Intensive Care Unit. *Significantly different group as per posthoc tests.

Factors	Area	N	μ	SD	F	p-value
							Circumstances of the handover	OR to PACU	38	2.75	0.79	1.761	0.158
PACU to SICU	25	2.5	0.76										
PACU to Surgical Wards	59	2.85	0.74										
Conducting the handover	OR to PACU	38	3.62	0.33	0.663	0.576
	PACU to SICU	25	3.72	0.31		
	PACU to Surgical Wards	59	3.7	0.33		
	PACU to Day Surgery Unit	21	3.68	0.38		
Teamwork	OR to PACU	38	3.63	0.38	2.076	0.106
	PACU to SICU	25	3.86	0.28		
	PACU to Surgical Wards	59	3.73	0.39		
	PACU to Day Surgery Unit	21	3.7	0.35		
Handover quality	OR to PACU	38	3.24	0.51	4.707	0.004
	PACU to SICU	25	3.15	0.35		
	PACU to Surgical Wards	59	3.08	0.35		
	PACU to Day Surgery Unit	21	3.47*	0.43		

Data, however, showed a significant statistical difference (p=0.004) concerning the handover quality according to the involved postoperative handover departments (from-to). Posthoc analyses were, therefore, carried out using the Bonferroni test. PACU to Day Surgery Unit handover showed a highly significant statistical handover quality mean difference compared to the rest of the units' transfer. The rest of the wards' handover process had similar perceptions of the quality of the handover (Table 6).

Nurses’ perception regarding the quality of postoperative handover practice based on selected demographic variables

As shown in Table 7, there was a significant statistical difference for the agreement on the handover quality (p=0.036), as well as circumstance of postoperative handover (p=0.046) based on respondents’ age. Nurses’ perceptions of conduct and teamwork during the postoperative handover did not differ based on age group. Moreover, significant statistical differences were found for the circumstance of handover (p=0.031), as well as teamwork (p=0.019) between the receiving and handing respondents; nurses’ receiving ratings were higher than the ratings of handing nurses.

**Table 7:** Nurses’ Rating of the Overall Postoperative Handover Quality Based on Participants' Demographic Characteristics μ=Mean; SD=Std. Deviation. *Significantly different group as per posthoc tests.

Factors		Circumstances of the handover	Conduct of the handover	Teamwork	Handover quality
		μ (SD)	μ (SD)	μ (SD)	μ (SD)
Age of the Nurse	24-30	2.53 (0.75)	3.57 (0.38)	3.64 (0.41)	3.04 (0.31)
	31-40	2.85 (0.76)	3.71 (0.30)	3.76 (0.33)	3.25 (0.43)
	>40	2.92 (0.56)	3.72 (0.34)	3.71 (0.44)	3.26 (0.54)*
	F	3.143	2.655	1.249	3.400
	p-value	0.046	0.074	0.290	0.036
Role During Handover	Transfer	2.60 (0.81)	3.64 (0.32)	3.62 (0.43)	3.26 (0.50)
	Receiving	2.88 (0.68)	3.70 (0.34)	3.78 (0.32)	3.16 (0.3)
	F	-2.17	-1.10	-2.40	1.32
	p-value	0.031	0.272	0.019	0.192
Total Professional Experience	1-5	2.39 (0.83)	3.51 (0.38)	3.64 (0.43)	2.99 (0.26)*
	6-10	2.86 (0.77)	3.76 (0.28)	3.66 (0.38)	3.20 (0.34)
	11-15	2.88 (0.70)	3.63 (0.39)	3.76 (0.38)	3.25 (0.52)
	16-20	2.65 (0.64)	3.75 (0.18)	3.79 (0.26)	3.02 (0.30)
	>20	2.89 (0.66)	3.76 (0.22)	3.80 (0.34)	3.51 (0.49)
	F	2.074	2.883	0.926	4.455
	p-value	0.088	0.174	0.451	0.021
Total Experience in the Present Unit	1-5	2.75 (0.71)	3.63 (0.38)	3.24 (0.34)	3.21 (0.47)
	6-10	2.86 (0.79)	3.73 (0.30)	3.23 (0.27)	3.04 (0.48)
	>10	2.61 (0.65)	3.71 (0.24)	3.40 (0.27)*	3.08 (0.56)
	F	0.884	1.457	3.391	2.044
	p-value	0.415	0.236	0.036	0.133
Educational Level	Diploma	2.82 (0.70)	3.67 (0.35)	3.67 (0.40)	3.21 (0.49)
	Bachelor or higher	2.76 (0.75)	3.68 (0.33)	3.74 (0.36)	3.19 (0.41)
	t-test	0.42	-0.12	-0.89	0.27
	p-value	0.678	0.902	0.374	0.788

Concerning total professional experience, a significant statistical difference was observed in the handover quality items based on respondents' experience. Finally, ratings for circumstances, conduct, teamwork, and handover quality did not differ based on respondents’ educational level or current nursing role.

Posthoc analyses were, therefore, carried out using the Bonferroni test for analyses that had omnibus (i.e., initial ANOVA) significant differences. As shown, nurses above 40 years of age had the highest rating for circumstances, as well as handover quality, compared to the rest of the other age groups. People who have five years of professional experience or less rated their quality of handover practice less compared to other groups. Finally, nurses who had more than 10 years of unit experience had the highest teamwork ratings.

Nurses’ perception regarding the quality of postoperative handover practice based on patients' status

As shown in Table 8, the ANOVA test revealed no significant statistical differences in nurses’ perception of the overall postoperative handover quality (p>0.05), as per patients’ age group. Concerning patients’ blood circulation and respiration, the perception of the circumstance of postoperative handover and handover quality was significantly different according to patients’ circulation and respiratory status (p=0.023, p=0.033, respectively).

**Table 8:** Differences in Nurses’ Perception of the Overall Postoperative Handover Quality Based on the Patient’s Status μ=Mean; SD=Std. Deviation. *Significantly different group as per posthoc tests.

Factors		Circumstances of the handover	Conduct of the handover	Teamwork	Handover quality
		μ (SD)	μ (SD)	μ (SD)	μ (SD)
Age of the patient	18-30	2.97 (0.73)	3.60 (0.39)	3.70 (0.37)	3.16 (0.40)
	31-40	2.74 (0.80)	3.69 (0.30)	3.66 (0.38)	3.29 (0.50)
	41-50	2.59 (0.71)	3.61 (0.37)	3.74 (0.37)	3.14 (0.45)
	51-60	2.85 (0.60)	3.76 (0.25)	3.78 (0.37)	3.26 (0.39)
	>60	2.85 (0.80)	3.74 (0.31)	3.74 (0.39)	3.10 (0.35)
	F	1.118	1.280	0.421	1.106
	p-value	0.351	0.281	0.793	0.356
Blood circulation and respiration	Impaired	2.25 (0.77)	3.65 (0.30)	3.92 (0.13)	3.17 (0.43)
	Medium	2.53 (0.58)	3.59 (0.42)	3.63 (0.49)	3.00 (0.31)
	Good	2.86 (0.75)*	3.70 (0.31)	3.73 (0.35)	3.24 (0.44)*
	F	3.893	1.274	1.759	3.485
	p-value	0.023	0.283	0.176	0.033
Level of consciousness	Unconscious	2.31 (0.56)	3.72 (0.29)	3.81 (0.31)	3.06 (0.30)
	Semiconscious	2.65 (0.74)	3.61 (0.33)	3.63 (0.40)	3.15 (0.45)
	Awake	2.90 (0.73)*	3.69 (0.34)	3.74 (0.37)	3.23 (0.44)
	F	5.227	0.854	1.539	1.382
	p-value	0.006	0.428	0.218	0.254
Experience of nausea or feeling unwell	No	2.79 (0.77)	3.70 (0.32)	3.76 (0.35)	3.23 (0.44)
	Yes	2.75 (0.71)	3.64 (0.35)	3.67 (0.40)	3.14 (0.41)
	F	0.297	1.073	1.487	1.221
	p-value	0.767	0.285	0.139	0.224
Pain perceptions	Moderate	2.70 (0.78)	3.61 (0.35)	3.69 (0.40)	3.20 (0.43)
	Slight	2.86 (0.65)	3.69 (0.30)	3.72 (0.33)	3.21 (0.45)
	No pain	2.75 (0.81)	3.76 (0.34)	3.76 (0.40)	3.17 (0.41)
	F	0.722	2.396	0.456	0.118
	p-value	0.487	0.095	0.635	0.889
Physical status classification per the American Society of Anesthesiology	Healthy	2.89 (0.84)	3.70 (0.34)	3.74 (0.37)	3.32 (0.47)*
	Mild disease	2.70 (0.66)	3.64 (0.34)	3.65 (0.40)	3.09 (0.38)
	Severe disease limits activity	2.75 (0.47)	3.74 (0.30)	3.81 (0.32)	2.98 (0.10)
	Severe disease threat to life	2.36 (0.63)	3.66 (0.34)	3.93 (0.12)	3.20 (0.37)
	F	1.476	.524	1.778	4.457
	p-value	0.224	0.666	0.154	0.005
Anesthetic Techniques Used	General	2.74 (0.75)	3.69 (0.31)	3.73 (0.35)	3.21 (0.43)
	Regional	2.88 (0.72)	3.69 (0.47)	3.78 (0.34)	3.20 (0.48)
	Spinal	3.10 (0.52)	3.55 (0.48)	3.58 (0.59)	3.00 (0.41)
	F	1.154	0.791	0.894	1.114
	p-value	0.318	0.456	0.411	0.331
Type of Surgery	Emergency	2.65 (0.80)	3.65 (0.39)	3.82 (0.31)	3.15 (0.36)
	Elective	2.80 (0.73)	3.68 (0.32)	3.70 (0.38)	3.20 (0.44)
	F	-0.815	-0.427	1.352	-0.486
	p-value	0.416	0.670	0.179	0.628

Furthermore, there was a significant statistical difference (p=0.006) in the agreement of the postoperative handover circumstances based on patients' level of consciousness. Neither patients' experience of nausea or feeling unwell, feeling pain, anesthetic techniques used, nor the type of surgery affected nurses’ perception of the overall postoperative handover quality (p>0.05). Regarding patients’ physical status classification, only handover quality significantly differed (p=0.005) based on the physical status classification of the patients as per the American Society of Anesthesiology.

Posthoc analyses further revealed that nurses’ perception of circumstances, as well as handover quality, were the best where patient blood circulation and respiration were classified as satisfactory. Patients who were awake as per the level of consciousness assessment perceived handover circumstances higher than when they had impaired consciousness. Finally, a healthy rating for patient physical classification as healthy affected nurses' high rating for the quality of the handover compared to the rest of the categories of physical status.

## Discussion

The purpose of this research is to assess the quality of postoperative handover practice among nurses, specifically circumstances, teamwork, the conduct of the handover, and their overall perceived quality. Overall, the results of the present study, as compared to previous similar research, have emphasized the significant role of effective handover practices in the nursing profession.

The study found a significant agreement regarding the circumstances of postoperative handover, which were well-perceived by both the receiving and transferring nurses. This finding is in line with previous findings by Mamalelala [1]. It is essential to recognize that the overall nurses’ perception of the circumstances of the postoperative handover could significantly contribute to handover quality. The study showed that factors associated with poor perception of postoperative handover circumstances included time pressure and the complexity of the patient’s health. Reine et al. [5] stated that time strain during the postoperative handover is considered a threat to the handover quality. In the same vein, Schramm’s circular model of communication emphasized that the communication process is influenced by environmental factors, such as workload, which causes time pressure [35]. Similarly, the important role of having enough time for a quality handover was reported by the majority of nurses as being one of the most important criteria for evaluating the conduct of the handover process. This result ties nicely with previous studies, wherein 88% of nurses agreed on the need for securing enough time to ensure that a satisfactory handover was provided [5].

The present study further revealed that the conduct of the postoperative handover was perceived well among the majority of study participants. Logical sequencing of the postoperative handover, in addition to the vital role of available documentation in structuring the handover, was significantly agreed upon by nurses. The importance of utilizing a structured and logical handover process has also been emphasized in the literature not only for the quality of such a process but also for patients' safety. Leonardsen et al. [36] and Rikos et al. [37] stated that implementing a structured tool of communication that is based on a logical workable process with some standard interdepartmental communication mechanisms is significant for improving the safety and quality of handover and ensuring the consistency and continuity of patient care.

Nurses further emphasized the important role of minimizing interruptions during the handover process as a means of improving the quality. Murray et al. [38] stated that among the key barriers that could hinder the handover transition process by nurses is the prevalence of interruptions in the environment during the transition of the patient care process. The present study further confirmed that high-quality communication and discussion between the involved nurses in the postoperative handover must accurately include all critical information about the patient's condition, the patient’s assessment, any suspected risks or complications, and possible further treatment or interventions. A similar pattern of results was obtained in studies conducted by Manser et al. [29] and Manser et al. [39]. Additionally, Jones et al. [13] emphasized that transferring practitioners must communicate the essential factors about the patient's condition and ongoing treatment interventions to the healthcare worker receiving the same.

Concerning teamwork, the majority of study participants positively perceived the team process and the members involved in the postoperative handover. Almost all participating nurses had agreed on both the easiness of establishing satisfactory contact at the beginning of the handover and the completion of the handover jointly by all team members. In accordance with the present results, a previous study conducted by Streeter et al. [40] demonstrated that the interaction between transferring and receiving nurses during handover should ensure satisfactory contact to exchange information, ask and answer questions, and achieve optimal communication. Additionally, participating nurses agreed upon the vitality of having no tension between the teams and having questions and ambiguities adequately resolved. These results are in line with those of a previous study by Kerr [41] and Nagpal et al. [11]. Nagpal et al. [11] stated that achieving teamwork during handover correlated with decreasing missed information and lowering the chance of error, which consequently could lead to high-quality handover. Thomson [42] contended that excellent teamwork between receiving and handing over nurses could enhance participants’ perception of handover quality.

Furthermore, the study evaluated postoperative handover quality, the fourth construct of the HQRF, which focuses on documentation, shared information, the patient’s experience, and self-rating of the handover quality. The percentage of nurses who agreed on the high quality of the overall handover was equally high among both handing over and receiving nurses. Similar results were reported by Reine et al. [5].

Study results further revealed that complete documentation, as well as careful consideration of the patient’s experience, showed the highest agreement for handover quality among study participants. This was consistent with the findings of Manser et al. [39] and Reine et al. [5]. Manser et al. [39] reported that 92% of the participating nurses agreed on the necessity to respect patients’ experience during handover. Moreover, according to Manser et al. [39] and Segall et al. [43], complete and precise patient documents are very important to give a clear picture of the patient to receiving team, which ensures continuity of care and the patient’s safety. In line with that, the results of this study also indicated that more than half of the participating nurses agreed that not too much information was given and asked for during the handover. In fact, providing the most relevant and important patient information is very detrimental to facilitating the communication process. A high-quality handover should, therefore, be characterized by the exchange and sharing of information between two parties in which they are able to ask and answer the questions needed for securing the continuity of quality patient care [40]. Such results support the idea that a focused handover that eliminates irrelevant and unnecessary information is more likely to result in the best patient care [40].

The study further explored the differences in perceptions of practice quality based on involved departments, nurses’ handover roles, nurse participants’ demographics, and patients’ status variables. Nurses’ perception of handover quality revealed a statistically significant difference based on the departments involved, specifically the self-rating of handover quality. This result is in line with the study conducted by Koirala et al. [44], who showed that nursing perception of handover quality depended significantly upon the type of nursing unit. Similarly, Reine et al. [5] reported that the difference in the nature of nurses’ clinical practice throughout hospital areas is playing an important role in the variation of nursing perceptions of handover quality. The study further showed that nurses conducting handover between PACU and DSU were more likely to agree with the handover quality item than other postoperative handover areas. A possible explanation for this might be the difference in duty hours, workload, patients’ census, duration of hospital stays, and severity of conditions between DSU and other surgical departments.

Transferring and receiving nurses’ assessment of the postoperative handover practice

Concerning nurses’ handover role, the study revealed a significant difference between the receiving and handing over nurses' rating of the postoperative circumstances, as well as postoperative teamwork perceptions. The ratings of receiving nurses for the postoperative handover were higher than the transferring nurses. In the current study, the transferring nurses are either OR or PACU staff. Hence, the current study's findings do not support previous studies in which the transferring team was more satisfied with the postoperative handover than those who were receiving the patient's responsibility [5,9]. The reason behind transferring nurses’ lower perception of the postoperative circumstances and teamwork is that they do handover different cases with various surgical procedures to different hospital departments, which might make it difficult for them to figure out the different expectations of the receiving staff that have various backgrounds and views as well. Furthermore, Petrovic et al. [19] cited that the PACU accepts different conditions and communicates to transfer their responsibilities to more than one department in the hospital, which might be responsible for their flexibility, predetermined expectations, and consequently easily achieved satisfaction compared to transferring nurses. 

On the other hand, nurses’ handover role revealed no significant effect on perceptions of handover conduct or its quality. These findings could be attributed to the fact that the KKUH is implementing a standardized handover process that controls nurses’ practice role variations. Mohammad [25] reported that utilizing standardized handover tools is a way to overcome the difference in quality perception between nurses. A standard handover process makes it easy for nurses to conduct the handover consistently and have similar views of the handover quality [38,45-47].

Quality of postoperative handover practice based on the selected demographic variables

The present study findings revealed that nurses' total professional experience, total experience in the present unit, and nurses’ age affect the overall handover quality assessment. This was inconsistent with Koirala et al. [44] and Nagammal et al. [24]. Furthermore, the study results indicated that the total years of nursing experience did not affect the perception of nurses' postoperative handover circumstances or their teamwork. This finding contradicts the findings of Reine et al. [5]. Moreover, Redley et al. [48] reported that the ability of nurses to manage and deal with teamwork and relationships during postoperative handover is correlated with their length of experience. Moreover, the current study found, however, a not statistically significant result: nurses with 6-10 and 16-20 or more years of nursing experience had a better perception of handover conduct compared to those with five years or less of nursing experience. In fact, more experienced nurses are better familiar with the handover process between different hospital units than inexperienced nurses who might miss some of the important information during handover due to their unfamiliarity.

Additionally, nurses’ total professional experience enhanced their perception of the quality of postoperative handover, including evaluation of documentation, shared information, patient experience, and self-rating of the handover quality. Nurses with professional experience of more than 20 years reported the highest quality of postoperative handover. This result may be explained in light of the cumulative experiences and information gained by expert nurses, in addition to their critical thinking skills, which are used in widening their knowledge about patients, through asking and answering questions during handover. This explanation is supported by Mcfetridge et al.'s [49] study which found that junior nurses were not asking any questions during handover because they did not recognize that there was missing information.

In line with previous results, the study showed that, as nurses' experience in the assigned unit increased, the perception of the self-rating of handover teamwork increased. Consistent with the present study findings, Schramm's circular model of communication emphasized that, during postoperative handover communication, the transfer, receipt, and understanding of information are affected by the common field of experience, which includes the experience duration in the specialty unit of nurses [35]. Hilligoss and Cohen [50] stated in their study that nurses have an excellent chance to learn from each handover process they are involved in and that experience is the best source to get lessons about the handover process.

Concerning participants’ age, nurses older than 40 years old had a higher perception of postoperative handover circumstances compared to younger nurses. In the same vein, the study found that, as nurses’ age increases, the perception of the quality of postoperative handover increases through the evaluation of documentation, shared information, patient experience, and the self-rating of handover quality. This finding, however, is contrary to previous studies [24,42,44].

Quality of postoperative handover practice based on patients’ status variables

The present study revealed three characteristics related to the patients’ condition that had a significant impact on nurses’ assessment of the overall handover quality, including the patient’s blood circulation and respiration, level of consciousness, and physical status classification, as per the American Society of Anesthesiology. This finding is supported by Reine et al. [5] and Streeter et al. [40] who showed that a patient’s status affects the postoperative handover quality and that nurses must, therefore, recognize and understand the patients’ needs and conditions.

A significant difference was obtained in postoperative handover circumstance items, as well as handover quality based on a patient’s blood circulation and respiration (good, medium, impaired). The study showed that nurses tend to agree with the handover circumstances and the quality construct when a patient has good blood circulation and respiration than when a patient had a medium or impaired status. These results might be attributed to the fact that the impaired circulation and respiration reflect the high complexity of the case that does take the greatest focus and highest attention from the nurses during the handover process. Nurses also tend to work under pressure and stress when handing over patients with impaired circulation and/or respiration. Reine et al. [5] emphasized the significant effect of the patient’s condition on the quality of the handover process and revealed that handing over or receiving patients with complex problems is correlated with lower handover quality. 

Concerning the patient’s level of consciousness, a significant difference in the agreement of circumstance items was found. It was illustrated that nurses who received or transferred awake postoperative patients tend to agree on the handover circumstance items as they relate to quality handover more than those who received or transferred unconscious patients. In fact, patients with impaired consciousness require the highest concern from nurses, as well as priority monitoring, to prevent patient harm by removing tubes themselves, their unwanted movement, or deficient handover information. Patients’ safety in the handover process is considered a high priority for nurses, and earlier studies, therefore, emphasized the adaptation of the handover process based on the current patient’s status and handover circumstances to ensure quality and safe care [5,51].

Finally, the physical status classification of the patient, as per the American Society of Anesthesiology, significantly influenced the agreement on the handover quality construct. According to Manser et al. [39], information about a patient’s medical history and chronic illness is significant for an obvious picture of the patient. This study revealed that postoperative nurses who received or transferred healthy postoperative patients were more likely to agree on the handover quality items than those who received or transferred patients with mild systematic disease and severe systematic disease that limits normal activity. This finding is consistent with previous studies, which showed that the handover process tends to be effective and easy if the patient was in good health [5].

Implications and recommendations

Based on the study findings, the researchers recommended that nursing administration and policymakers have to continually evaluate the postoperative handover practice and understand the factors affecting the quality of the postoperative handover to decide and implement the appropriate interventions. It is also recommended to develop guidelines for postoperative handover to benefit from implementing the standardized handover process and the structured handover tool and enhancing nurses’ compliance. Planning and implementing postoperative handover training programs and workshops to enhance nurses’ communication and teamwork skills is necessary. 

Although the postoperative handover quality is perceived as high, repeating the study using qualitative research design, observation, and video or audio records with a larger sample will be needed to gain in-depth data about postoperative handover quality and find other possible affecting variables. Replication of the current study in other institutions, hospitals, and regions of Saudi Arabia to get a wide perspective on the postoperative handover and enhance the study findings' generalization. Finally, future studies are needed to investigate and bring out the relationship between postoperative handover quality and patient outcome.

Limitations

The present study has the following limitations. They first conducted this study in only one tertiary hospital in Riyadh due to the COVID-19 crisis and strict precautions. This, in turn, limits the generalization of the current study findings. Second, the participating nurses provided the survey questionnaire after they have completed the handover process. However, assessing a process, in which nurses are active stakeholders and using the self-report questionnaire, may not represent the reality of postoperative handover practice. Finally, using a sample size that considers relatively small and using convenience sampling limits the generalization of the findings.

## Conclusions

This research revealed that the quality of overall postoperative handover was high as perceived by handing over and receiving nurses. The postoperative handover was conducted well under satisfactory and acceptable circumstances, with ideal teamwork and high-rated quality. Involved departments (from-to) affected the nurses' perception of the overall postoperative handover quality, as shown in their ratings for the different handover quality items. This study found three nurses' characteristics that impacted handover quality assessment, including total professional experience, total experience in the present department, and nurses’ age. Additionally, there were three characteristics related to the patient's condition that also had an impact on the nurses’ assessment of the overall handover quality, which was the patient's blood circulation and respiration, the patient's level of consciousness, and the physical status classification of the patient as per the American Society of Anesthesiology. This study strongly emphasized the need to understand nurses' perceptions and satisfaction with the current postoperative handover practice for leaders and policymakers in the healthcare system to employ necessary policies and procedures that could enhance the current practice and promote patient care delivery.

## References

[1] Mamalelala TT (2023). Mamalelala TT. https://wiredspace.wits.ac.za/items/6625f38b-3d66-4f55-89e9-5d0162d92301.

[2] (2018). NICE: Structured patient handovers. https://www.nice.org.uk/guidance/ng94/evidence/32structured-patient-handovers-pdf-172397464671.

[3] (2020). WHO: Communication during patient handovers. https://cdn.who.int/media/docs/default-source/patient-safety/patient-safety-solutions/ps-solution3-communication-during-patient-handovers.pdf.

[4] Beuken JA, Verstegen DM, Dolmans DH (2020). Going the extra mile—cross-border patient handover in a European border region: qualitative study of healthcare professionals' perspectives. BMJ Qual Saf.

[5] Reine E, Ræder J, Manser T, Småstuen MC, Rustøen T (2019). Quality in postoperative patient handover: different perceptions of quality between transferring and receiving nurses. J Nurs Care Qual.

[6] Randmaa M, Engström M, Swenne CL, Mårtensson G (2017). The postoperative handover: a focus group interview study with nurse anaesthetists, anaesthesiologists and PACU nurses. BMJ Open.

[7] van der Wulp I, Poot EP, Nanayakkara PW, Loer SA, Wagner C (2019). Handover structure and quality in the acute medical Assessment Unit: a prospective observational study. J Patient Saf.

[8] Puzio TJ, Murphy PB, Virtanen P, Harvin JA, Hartwell JL (2020). Handover practices in trauma and acute care surgery: a multicenter survey study. J Surg Res.

[9] Smeulers M, Lucas C, Vermeulen H (2014). Effectiveness of different nursing handover styles for ensuring continuity of information in hospitalised patients. Cochrane Database Syst Rev.

[10] Fahim Yegane SA, Shahrami A, Hatamabadi HR, Hosseini-Zijoud SM (2017). Clinical information transfer between EMS staff and emergency medicine assistants during handover of trauma patients. Prehosp Disaster Med.

[11] Nagpal K, Abboudi M, Manchanda C (2013). Improving postoperative handover: a prospective observational study. Am J Surg.

[12] Singh H, Thomas EJ, Petersen LA, Studdert DM (2007). Medical errors involving trainees: a study of closed malpractice claims from 5 insurers. Arch Intern Med.

[13] Jones PM, Cherry RA, Allen BN (2018). Association between handover of anesthesia care and adverse postoperative outcomes among patients undergoing major surgery. JAMA.

[14] Al Wahabi S, Farahat F, Bahloul AY (2017). Prevalence and preventability of sentinel events in Saudi Arabia: analysis of reports from 2012 to 2015. East Mediterr Health J.

[15] Spooner AJ, Aitken LM, Corley A, Chaboyer W (2018). Developing a minimum dataset for nursing team leader handover in the intensive care unit: a focus group study. Aust Crit Care.

[16] Kitney P, Tam R, Bennett P, Buttigieg D, Bramley D, Wang W (2016). Handover between anaesthetists and post-anaesthetic care unit nursing staff using ISBAR principles: a quality improvement study. ACORN.

[17] Horwitz LI, Dombroski J, Murphy TE, Farnan JM, Johnson JK, Arora VM (2013). Validation of a handoff assessment tool: the Handoff CEX. J Clin Nurs.

[18] Clarke S, Clark-Burg K, Pavlos E (2018). Clinical handover of immediate post-operative patients: a literature review. J Perioper Nurs.

[19] Petrovic MA, Aboumatar H, Scholl AT (2015). The perioperative handoff protocol: evaluating impacts on handoff defects and provider satisfaction in adult perianesthesia care units. J Clin Anesth.

[20] Joint Commission. (2009 (2023). Joint Commission International: Communicating clearly and effectively to patients. https://store.jointcommissioninternational.org/assets/3/7/jci-wp-communicating-clearly-final_(1).pdf.

[21] Joint Commission (2012). Joint Commission Center for Transforming Healthcare releases targeted solutions tool for hand-off communications. Jt Comm Perspect.

[22] Joint Commission (2023). Sentinel event alert: Complimentary publication of the Joint Commission International. https://www.jointcommission.org/-/media/tjc/documents/resources/patient-safety-topics/sentinel-event/sea_58_hand_off_comms_9_6_17_final_(1).pdf.

[23] Weinger MB, Slagle JM, Kuntz AH (2015). A multimodal intervention improves postanesthesia care unit handovers. Anesth Analg.

[24] Nagammal S, Nashwan AJ, Nair SL, Susmitha A (2016). Nurses’ perceptions regarding using the SBAR tool for handoff communication in a tertiary cancer center in Qatar. J Nurs Educ Pract.

[25] Mohammad A (2017). A discourse analysis of nursing handoffs: exploring nurse-to-nurse interactions in two hospitals in Saudi Arabia. USF Tampa Graduate Theses and Dissertations.

[26] Suganandam DK (2018). Handoff communication: hallmark of nurses. Indian Journal of Continuing Nursing Education.

[27] Mohamed A (2017). Effectiveness of handoff educational program on nurses’ interns’ knowledge, and communication competence. Am J Nurs.

[28] Park M, Park J (2016). Qualitative versus quantitative research methods: discovery or justification. J Mark Thought.

[29] Manser T, Foster S, Gisin S, Jaeckel D, Ummenhofer W (2010). Assessing the quality of patient handoffs at care transitions. Qual Saf Health Care.

[30] Asman O, Melnikov S, Barnoy S, Tabak N (2019). Experiences, behaviors, and perceptions of registered nurses regarding research ethics and misconduct. Nurs Ethics.

[31] Petillion W, Melrose S, Moore SL, Nuttgens S (2017). Graduate students’ experiences with research ethics in conducting health research. Research Ethics.

[32] Miracle VA (2016). The Belmont Report: the Triple Crown of research ethics. Dimens Crit Care Nurs.

[33] Sellman D (2016). The practice of nursing research: getting ready for 'ethics' and the matter of character. Nurs Inq.

[34] Obeidat RF, Al-Delaimy W (2020). Applying the ethical principle of social benefits in nursing research in developing countries: the case of Jordan. J Acad Ethics.

[35] Arnold EC, Boggs KU (2019). Interpersonal Relationships E-Book: Professional Communication Skills for Nurses. https://books.google.com.sa/books/about/Interpersonal_Relationships_E_Book.html?id=HU1PAQAAQBAJ&redir_esc=y.

[36] Leonardsen AC, Moen EK, Karlsøen G, Hovland T (2019). A quantitative study on personnel’s experiences with patient handovers between the operating room and the postoperative anesthesia care unit before and after the implementation of a structured communication tool. Nursing Reports.

[37] Rikos N, Linardakis M, Merkouris A, Rovithis M, Philalithis A (2019). Exploration of the experiences, opinions and attitudes of nurses on inter-shift handover procedures. Archives of Hellenic Medicine/Arheia Ellenikes Iatrikes.

[38] Murray JS, McGrath J, Smith MF (2013). Understanding the clinical handoff perspective of pediatric emergency nurses. Pediatr Nurs.

[39] Manser T, Foster S, Flin R, Patey R (2013). Team communication during patient handover from the operating room: more than facts and figures. Hum Factors.

[40] Streeter AR, Harrington NG, Lane DR (2015). Communication behaviors associated with the competent nursing handoff. J Appl Commun.

[41] Kerr MP (2002). A qualitative study of shift handover practice and function from a socio-technical perspective. J Adv Nurs.

[42] Thomson H, Tourangeau A, Jeffs L, Puts M (2018). Factors affecting quality of nurse shift handover in the emergency department. J Adv Nurs.

[43] Segall N, Bonifacio AS, Schroeder RA (2012). Can we make postoperative patient handovers safer? A systematic review of the literature. Anesth Analg.

[44] Koirala S, Mehta RK, Acharya S, Gauro P (2019). Critical care nurses' views on handover in Chitwan, Nepal. CONNECT: The World of Critical Care Nursing.

[45] Randmaa M, Mårtensson G, Swenne CL, Engström M (2015). An observational study of postoperative handover in anesthetic clinics; the content of verbal information and factors influencing receiver memory. J Perianesth Nurs.

[46] Naour MG (2018). Transition of care: the evaluation of hand-off communication between emergency department and medical/Surgical nursing units. Doctoral Dissertation. ProQuest Dissertations & Theses Global.

[47] Kilic SP, Nimet Ovayolu RN, Ozlem Ovayolu RN, Ozturk MH (2017). The approaches and attitudes of nurses on clinical handover. Int J Caring Sci.

[48] Redley B, Bucknall TK, Evans S, Botti M (2016). Inter-professional clinical handover in post-anaesthetic care units: tools to improve quality and safety. Int J Qual Health Care.

[49] McFetridge B, Gillespie M, Goode D, Melby V (2007). An exploration of the handover process of critically ill patients between nursing staff from the emergency department and the intensive care unit. Nurs Crit Care.

[50] Hilligoss B, Cohen MD (2011). Hospital handoffs as multifunctional situated routines: implications for researchers and administrators. Adv Health Care Manag.

[51] Gu X, Liu HC, Itoh K (2019). Inter-department patient handoff quality and its contributing factors in Chinese hospitals. Cognition, Technology & Work.

